# Correction: Human observers have optimal introspective access to perceptual processes even for visually masked stimuli

**DOI:** 10.7554/eLife.16332

**Published:** 2016-03-31

**Authors:** Megan AK Peters, Hakwan Lau

Peters MAK, Lau H. 2015. Human observers have optimal introspective access to perceptual processes even for visually masked stimuli. *eLife* **4**:e09651. doi: 10.7554/eLife.09651.

Published October 03, 2015

In the published article, the code file used to generate the bottom row of Figure 3 contained an error. This led to altered visualization of the summary statistics for the model’s behaviour. The error was not present in the code file used to fit the model to behavioural data or calculate its goodness of fit, and so does not affect any quantitative metrics presented in the article. Additionally, due to an oversight the source code for the ideal observer model was not included in the originally published article. The correct code is now available.

The corrected Figure 3 is shown here:

**Figure:**
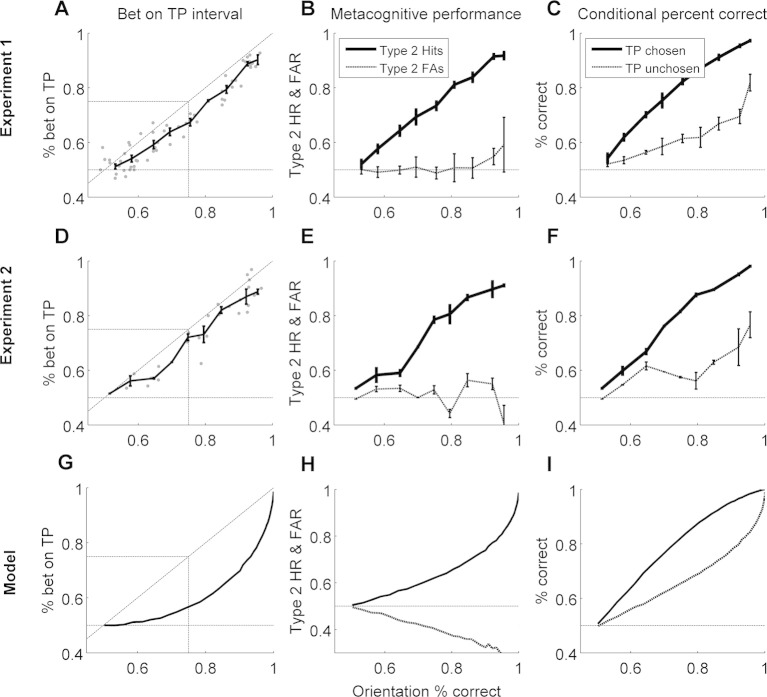

The originally published Figure 3 is shown for reference:

**Figure:**
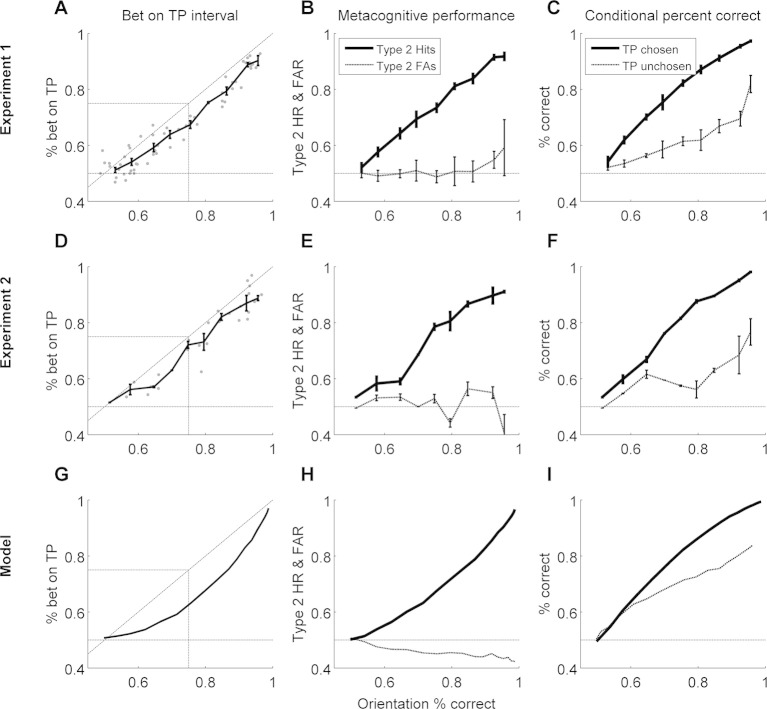

The article has been corrected accordingly.

